# Effect of Soda Residue Addition and Its Chemical Composition on Physical Properties and Hydration Products of Soda Residue-Activated Slag Cementitious Materials

**DOI:** 10.3390/ma13071789

**Published:** 2020-04-10

**Authors:** Yonghui Lin, Dongqiang Xu, Xianhui Zhao

**Affiliations:** 1 School of Civil and Transportation Engineering, Hebei University of Technology, Tianjin 300401, China; 2002021@hebut.edu.cn (D.X.); 201411601006@stu.hebut.edu.cn (X.Z.); 2 Department of Economics and Management, Hebei Normal University for Nationalities, Chengde 067000, China

**Keywords:** alkaline activation, soda residue, ground granulated blast furnace slag, physical properties, hydration products, microstructure

## Abstract

Soda residue (SR), the solid waste of Na_2_CO_3_ produced by ammonia soda process, pollutes water and soil, increasing environmental pressure. SR has high alkalinity, and its main components are Ca(OH)_2_, NaCl, CaCl_2_, CaSO_4_, and CaCO_3_, which accords with the requirements of being an alkali activator. The aim of this research is to investigate the best proportion of SR addition and the contribution of individual chemical components in SR to SR- activated ground granulated blast furnace slag (GGBS) cementitious materials. In this paper, GGBS pastes activated by SR, Ca(OH)_2_, Ca(OH)_2_ + NaCl, Ca(OH)_2_ + CaCl_2_, Ca(OH)_2_ + CaSO_4_, and Ca(OH)_2_ + CaCO_3_ were studied regarding setting time, compressive strength (1 d, 3 d, 7 d, 14 d, 28 d), hydration products, and microstructure. The results demonstrate that SR (24%)-activated GGBS pastes possess acceptable setting time and compressive strength (29.6 MPa, 28 d), and its hydration products are calcium silicate hydrate (CSH) gel, calcium aluminum silicate hydrates (CASH) gel and Friedel’s salt. CaCl_2_ in SR plays a main role in hydration products generation and high compressive strength of SR- activated GGBS pastes.

## 1. Introduction

With the rapid development of the construction industry, concrete has become the most widely used construction materials in the world, which leads to the increase of cement production year by year [1,2]. Studies show that the production of 1 ton of cement generates 0.94 tons of CO_2_. In 2018, global cement production reached 3.95 billion tons, that is to say, it adds about 3.71 billion tons of CO_2_ to the atmosphere, equivalent to 5% to 7% of the global total [1,3,4,5,6]. To solve this environmental problem, waste/by-product materials, such as fly ash and GGBS, have been used for the formation of alkali-activated cementitious materials as an alternative to cement [5,7].

GGBS, as a by-product material obtained during the manufacture of pig-iron, is produced in large amount around the world. The main chemical components of GGBS are CaO, SiO_2_, and Al_2_O_3_ [5]. Numerous studies show that alkali-activated GGBS (AAS) cementitious materials have excellent properties such as high strength, corrosion resistance, low porosity, frost resistance, low permeability, fire resistance, solidification of heavy metals, and are considered as low carbon environmental friendly cementitious materials [8,9,10].

For AAS pastes, the most used alkaline activators are NaOH or KOH with Na_2_SiO_3_⋅nH_2_O or K_2_SiO_3_⋅nH_2_O [11]. Researchers found that using them as activators can have impacts on environment, including human toxicity, fresh water and marine eco toxicity [12,13]. Some researchers also found that these AAS pastes require lots of alkali-activators, which may lead to over-rapid setting, strength shrinkage of AAS pastes, and uneconomical efficiency [12,13,14,15]. In recent years, scholars have begun to explore the use of alkaline waste as an alkali-activator, such as carbide slag [16,17,18], and SR [19,20,21,22,23].

Sodium carbonate (Na_2_CO_3_), also known as soda ash, is an important chemical raw material widely used in modern industries [24,25]. SR, the solid waste of Na_2_CO_3_ produced by Solvay process, contains mostly soluble matter (CaCl_2_, NaCl) and suspended matter (CaCO_3_, CaSO_4_, Ca(OH)_2_, SiO_2_, MgCO_3_, Al_2_O_3_ and Fe_2_O_3_) [26]. One kg of Na_2_CO_3_ produced by Solvay process generates 0.30 to 0.60 kg (depending on the composition of the raw materials) of SR [24,26,27,28]. China produced about 26.20 million tons of Na_2_CO_3_ in 2018 [29], and more than 60% of the Na_2_CO_3_ is produced by Solvay process [24]. That is, China produced at least 4.72 million tons of SR in 2018, resulting in a large quantity of SR piled up together, occupying lots of land. Its high alkalinity (pH value is 10–12) and chemical compositions pollute water and soil, causing environmental pressure and safety risks [23,25].

Numerous studies were carried out to utilize SR for stabilizing heavy metal [28,30], soil improvement [21], and recovery of calcium carbonate [25,31], but the utilization of SR was limited compared to the output of SR. Some studies used SR as a raw material for cement production, which was still not widely used due to the influence of high chloride concentration in SR [27,32]. Several studies found that AAS cementitious materials could solidify chloride ions. Cheng, Y. et al. [33] added NaCl and CaCl_2_ to NaOH-activated GGBS cementitious materials, and found that GGBS could combine with chloride ions to form calcium chloroaluminate hydrate (Fs salt, Friedel’s salt). Luoa, R. et al. [34] studied the chloride binding in GGBS concrete, and found that the output of Fs salt formed by GGBS was much more than ordinary Portland cement.

Several studies utilized SR to prepare geopolymers or cementitious materials [20,23,26,35], and revealed the possibility of SR reacting with solid alumosilicate materials (fly ash, GGBS or fly ash + GGBS) to form CSH and CASH gels under the use of alkaline activators (NaOH, Na_2_SiO_3_). Zhao, X. et al. [20] investigated the use of SR with fly ash under 8 mol/L NaOH in a geopolymer mortar. In their studies, by replacing 20% fly ash with SR, the compressive strength could reach 14.5 and 18.0 MPa, respectively, after being sealed and curing for 60 and 180 days, under room temperature, due to the coexistence of CSH gel, CASH gel, as well as sodium aluminosilicate polymer (N-A-S-H) or calcium-containing sodium aluminosilicate (C, N-A-S-H) gel. Sun and Gu [35], taking SR, GGBS, and fly ash as main raw materials, added 12% gypsum and 5% alkaline activator (Na_2_SiO_3_), to prepare cementitious materials, the flexural and compressive strength of which could reach 6.4 and 35.2 MPa, respectively, in 28 days.

As can be seen from the above studies, SR was used to prepare geopolymers or cementitious materials, but it needed to be used with alkaline activators (NaOH, Na_2_SiO_3_). The aim of this study is to use SR alone to activate GGBS and investigate the effect of the chemical compositions of SR on the slags. Firstly, this study investigates the development of setting time and compressive strength of SR-activated GGBS pastes at various SR/GGBS ratios. Secondly, 24% SR is selected to calculate the amount of each chemical component in SR, and to analyze the effect of each component on setting time and compressive strength. Finally, the hydration products of SR (24%) and its components are analyzed respectively when activating GGBS using X-ray diffraction spectroscopy (XRD), scanning electron microscopy (SEM), and Fourier transformed infrared spectroscopy (FTIR) techniques. The obtained results would provide theoretical basis for the use of SR as an activator, and benefit the future applications of SR in AAS cementitious materials.

## 2. Materials and Methods

### 2.1. Materials

#### 2.1.1. SR

**Figure 1:**
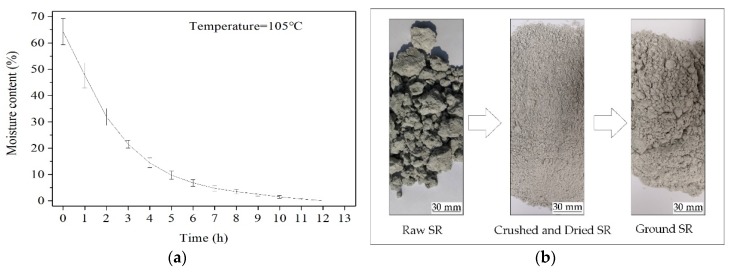
Moisture content of SR (**a**) and flow chart of SR pretreatment (**b**).

**Figure 2:**
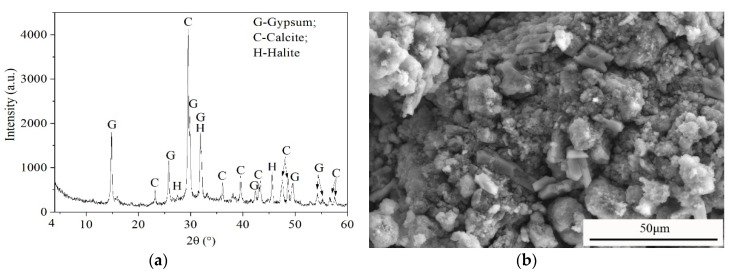
XRD pattern (**a**) and SEM image (**b**) of SR.

The SR used in this study was obtained from Tangshan Sanyou Chemical Industries Company Limited of Tangshan, Hebei Province, China. The chemical compositions of SR measured by X-ray fluorescence (XRF) technique are listed in Table 1. The raw SR has 60% to 70% moisture content, as shown in Figure 1a. In this study, the raw SR was first crushed and dried in an oven at 105 °C for 12 h, and then ground to a particle size less than 0.075 mm, as shown in Figure 1b. The average pH value of ground SR at 100% water content, at 25 ± 1 °C, was 12. The XRD pattern and SEM image of the ground SR are provided in Figure 2. It can be seen that the SR is composed of gypsum, calcite and halite phases, and the structure of SR is loose and porous.

#### 2.1.2. GGBS

**Figure 3:**
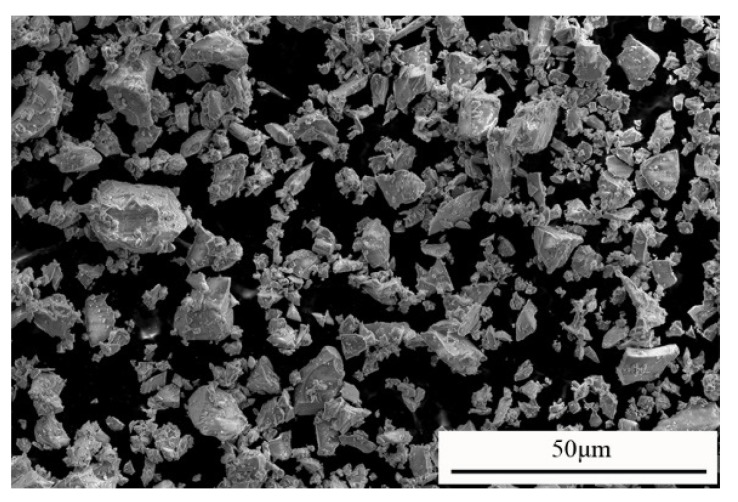
SEM image of GGBS.

The GGBS used in this study was obtained from a Slag Powder Company Limited in Tangshan, Hebei Province of China. The chemical compositions of GGBS measured by XRF technique are listed in Table 1. The main chemical components of the GGBS are Ca(OH)_2_, SiO_2_, and Al_2_O_3_, accounting for 84.3% of the total, which indicates that it can react with alkaline substances to produce cementitious materials. The measured specific gravity, the average diameter and the specific surface area of GGBS are 2.87, 12.7 μm and 450 m^2^/kg, respectively. It can be seen from the SEM image of GGBS provided in Figure 3 that the surface of GGBS is smooth, with clear edges and corners and without agglomeration.

#### 2.1.3. Chemicals

Reagent-grade Ca(OH)_2_, NaCl, CaCl_2_, CaSO_4_, and CaCO_3_, were obtained from Tianjin, China.

### 2.2. Methods

#### 2.2.1. Preparation of Specimens

In this study, nine groups of alkali-activated GGBS (AAS) cementitious materials were prepared, including four SR-activated GGBS pastes, made by increasing the proportion of SR in the pastes (SR + GGBS) from 8 to 32 wt.%, and another five AAS pastes (Ca(OH)_2_-activated GGBS, Ca(OH)_2_ + NaCl-activated GGBS, Ca(OH)_2_ + CaCl_2_-activated GGBS, Ca(OH)_2_ + CaSO_4_-activated GGBS, and Ca(OH)_2_ + CaCO_3_-activated GGBS pastes). It is worth noting that GGBS cannot be activated by NaCl, CaCl_2_, CaSO_4_, or CaCO_3_, without an alkaline activator. In this study, quantitative Ca(OH)_2_ in SR was used to provide an alkaline environment. 24% SR was selected to calculate the mass of Ca(OH)_2_, NaCl, CaCl_2_, CaSO_4_ and CaCO_3_, using Equation (1):

(1) $$M_{X}=M_{\mathrm{SR}}\times X\%$$

where X is Ca(OH)_2_, NaCl, CaCl_2_, CaSO_4_ or CaCO_3_; M_SR_ and M_X_ are the mass of SR (24% addition) and X, respectively; X% is the percentage of X in Table 1.

The calculation results and the mixing proportions of AAS cementitious materials are shown in Table 2. The water-to-solid ratio is 0.5, where solid is defined as the mixture of GGBS and activators solid. According to the proportion in Table 2, solid powders were firstly mixed and homogenized for 3 min in a laboratory mixer, then water was added and the mixing continued for another 3 min. Thereafter, the homogenized paste was then placed in a steel mold (40 mm × 40 mm × 160 mm) with two layers according to GB/T 17671-1999 (ISO) standard (China) [36]. Each layer of the mixture was vibrated for 1 min on the vibrating table. The prepared specimens were firstly placed in sealed plastic bags, cured at room temperature (25 ± 3 °C) for 24 h, and then demoulded, sealed and further cured under the same condition. After the curing (1 d, 3 d, 7 d, 14 d, and 28 d), compressive strength tests were performed on the specimens. The crushed samples were immersed in ethanol for 7 days to stop hydration. After being dried in a vacuum at 60 °C for 8 h, they were used for the measurements of SEM. In addition, the small pieces were ground to pass through a 0.075 mm sieve for the measurements of XRD and FTIR.

#### 2.2.2. Setting Time

The setting time of AAS pastes was determined by the Vicat apparatus according to GB/T 1346-2011 standard (China) [37]. AAS pastes were prepared, mixed and tested at 20 ± 2 °C, RH (relative humidity) ≥ 50%, and the specimens were curing at 20 ± 2 °C, RH ≥ 90%. The initial setting time is from the time of adding water to the time when the test needle sinks to 4 ± 1 mm away from the glass plate. The final setting time is the time from adding water to the time when the test needle sinks into the test body 0.5 mm.

#### 2.2.3. Compressive Strength

After the curing (1 d, 3 d, 7 d, 14 d, and 28 d) of AAS pastes, compressive strength test was performed on the specimens. A servo-control testing machine with loading capacity of 300 kN was employed. According to GB/T 17671-1999 (ISO) standard (China) [36], the compression area was 40 mm × 40 mm, and the loading rate was 2.4 kN/s. For each group, compressive strength was measured and averaged from six specimens.

#### 2.2.4. Phase Analysis and Microstructure

XRD test: The samples for XRD tests were taken from raw SR, raw GGBS, and S3, S5–S9 at 28 d. The XRD patterns were recorded on a Rigaku D/Max-2500 X-ray diffractometer (Akishima, Tokyo, Japan) with CuKα radiation using a generator voltage of 40 kV and a generator current of 150 mA, at a scanning rate of 4°/min, over a 2θ angular range from 4° to 60°.

SEM test: The microstructure of raw SR, raw GGBS, S3, S5–S9 were observed by SEM (Quanta FEG450, Hillsboro, OR, USA). Images were taken at 5 kV, 10000×.

FTIR test: Bonding characteristics of raw SR, raw GGBS, S3, S5, S7 and S8 were analyzed with a FTIR (Tensor 27, Bruker, Karlsruhe, Germany). The samples were ground and weighed 1.3 ± 0.001 mg with 130 mg KBr pellets to prepare tested specimens by mixing and pressing. The wavenumber ranged from 400 to 4000 cm^−1^.

## 3. Results and Discussion

### 3.1. Setting Time

**Figure 4:**
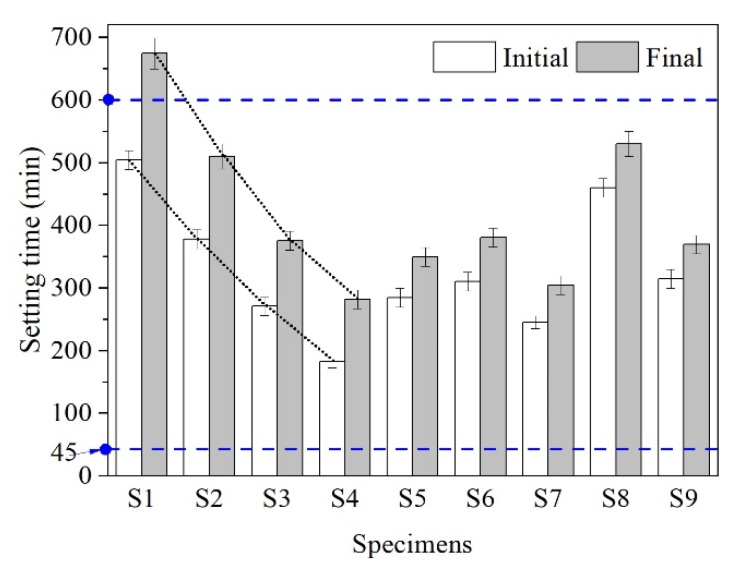
The setting time of AAS specimens: S1–S4, SR (8%, 16%, 24%, 36%)-activated GGBS pastes; S5, Ca(OH)_2_-activated GGBS pastes; S6, Ca(OH)_2_ + NaCl- activated GGBS pastes; S7, Ca(OH)_2_ + CaCl_2_-activated GGBS pastes; S8, Ca(OH)_2_ + CaSO_4_-activated GGBS pastes; S9, and Ca(OH)_2_ + CaCO_3_-activated GGBS pastes.

The purpose of setting time (initial, final) test is to investigate the working performance of AAS cementitious materials. Setting time of AAS pastes is presented in Figure 4. In this study, the setting time standard was according to OPC GB175-2007 standard (China) [38]; the initial setting time should be no less than 45 min, and the final setting time should be no more than 600 min.

As can be observed from Figure 4 (S1–S4), the setting time of SR-activated GGBS pastes varies greatly with different proportion of SR. Setting time of SR-activated GGBS pasts decreases with the increasing SR replacement. The final time of specimen S1 is 674 min, which is higher than the standard required value. The initial setting time of S2, S3, and S4 is found to be reduced by 25%, 46%, and 64%, respectively, and the final setting time is reduced by 24%, 44%, and 58%, respectively, compared to which of S1. The higher the proportion of SR is, the shorter the setting time is. As shown in Figure 4 (S5–S9), the setting time of AAS pastes varies with different activators. When Ca(OH)_2_ is used as GGBS activator alone, the initial and final setting time are 284 and 349 min, respectively. Further adding NaCl, CaSO_4_, or CaCO_3_ activators prolongs the setting time of AAS pastes, while adding CaCl_2_ activator shortens the setting time. As can be seen, among the main chemical compositions of SR, Ca(OH)_2_ + CaCl_2_ plays a major role in shortening the setting time of SR (24%)-GGBS pastes. The setting time is shortened with the increasing proportion of SR, which may be because Ca(OH)_2_ and CaCl_2_ in the solution increase. The increasing amount of Ca^2+^ ions in the solution accelerates the hydration reaction of GGBS pastes and the formation of CSH gel, causing the shortening of the setting time.

### 3.2. Compressive Strength

**Figure 5:**
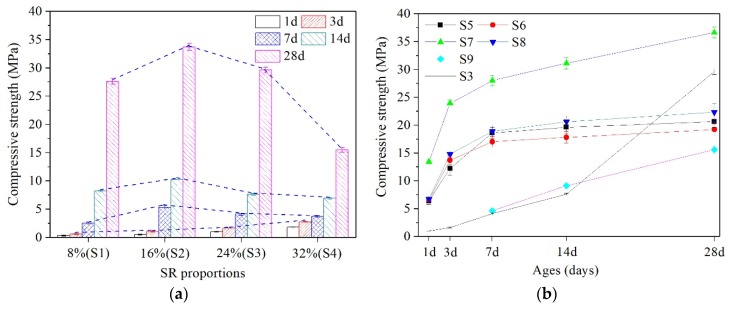
Compressive strength of AAS pastes: (**a**) S1–S4; (**b**) S3, S5–S9.

Figure 5 shows the compressive strength testing results (at the age of 1 d, 3 d, 7 d, 14 d, and 28 d) of the AAS cementitious materials. It can be seen that the compressive strength of AAS pastes increases as the curing time increases.

Compressive strength of AAS pastes with different proportions of SR are shown in Figure 5a. It can be clearly seen that the compressive strength of the SR (8% to 32%)-activated GGBS pastes at the age of 28 d is much higher than that at the ages of 7 d and 14 d. The early compressive strength (3 d) increases as SR proportion increases (from 8% to 32%), and the maximum value is 2.7 MPa, while the compressive strength from 7 d to 28 d increases as SR proportion increases to 16%, but decreases thereafter. The 28 d compressive strength (27.6, 33.7, 29.6 MPa) of SR (8% to 24%)-activated pastes varies a little. It is worth noting that when the proportion reaches 32%, the compressive strength (15.5 MPa) decreases significantly. Obviously, 8% to 24% SR addition gives acceptable to the compressive strength of the pastes at 28 d, while higher proportion of SR (32%) addition does not. By analyzing the setting time and the compressive strength, it can be concluded that 16% to 24% SR addition ensures the working performance and high strength of the pastes. In this study, 24% of SR addition was selected for comparative study in order to maximize the utilization of the solid waste, SR.

The development of the compressive strength of AAS pastes with different activators (24% SR, Ca(OH)_2_, Ca(OH)_2_ + NaCl, Ca(OH)_2_ + CaCl_2_, Ca(OH)_2_ + CaSO_4_, Ca(OH)_2_ + CaCO_3_) is shown in Figure 5b. It can be seen that the compressive strength varies with different activators. When Ca(OH)_2_ works as an activator, compressive strength of S5 at 7 d (18.6MPa) reaches 90% of that at 28 d (20.6 MPa), which indicates that the early compressive strength of S5 develops rapidly. Compared with Ca(OH)_2_, Ca(OH)_2_ + NaCl as an activator is conductive to the early (3 d) strength of S6, but had little effect on the strength thereafter; Ca(OH)_2_ + CaSO_4_ has a slight effect on improving the strength of S8; S7 had the highest strength at all ages, and the 28 d strength (36.6 MPa) is nearly 1.8 times than that of S5, which is similar to the research of Yum [39]. It is worth noting that the S9 has no measurable compressive strength at 3 d, which indicates that CaCO_3_ is the main reason for the low early strength of SR-activated GGBS pastes, as shown in Figure 5a. What is more, compared with the strength of S3, Ca(OH)_2_, NaCl, CaCl_2_ and CaSO_4_ in SR are conducive to the development of the early strength of the pastes, among which Ca(OH)_2_ + CaCl_2_ plays a major role.

### 3.3. XRD

**Figure 6:**
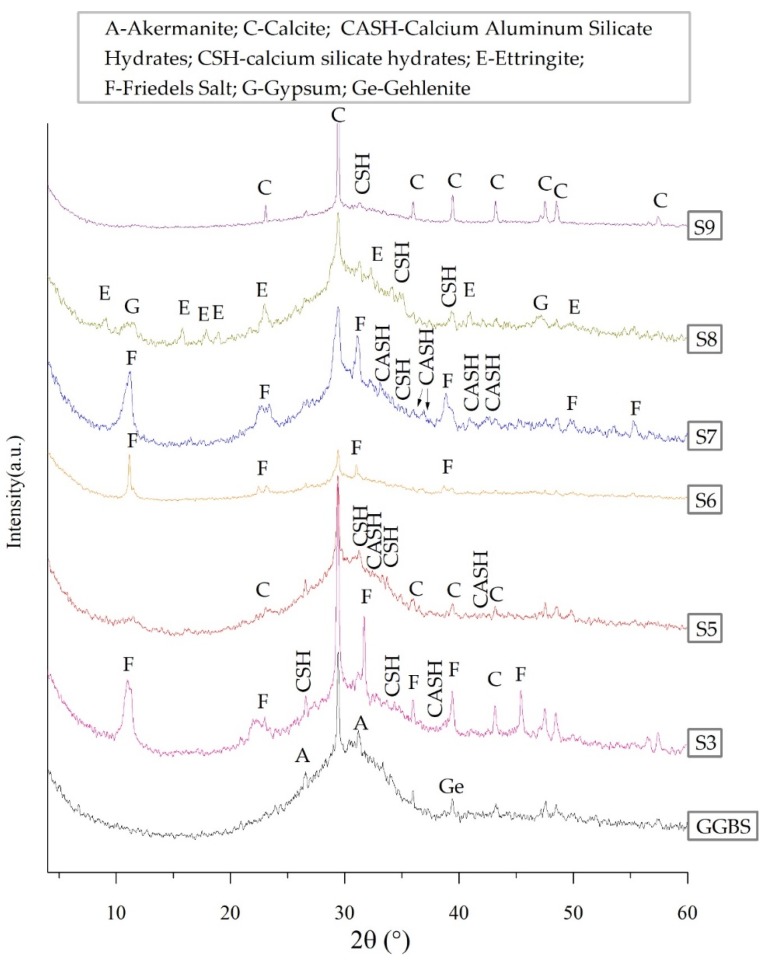
XRD patterns of raw GGBS and AAS pastes (S3, S5, S6, S7, S8, and S9) at 28 d.

The compressive strength of AAS pastes is directly affected by the hydration products. The XRD patterns of raw GGBS and AAS pastes (S3, S5, S6, S7, S8, S9) at 28 d are presented in Figure 6 with the crystalline phases. As shown in Figure 6, in the region of 20 to 40° 2θ, the raw GGBS shows an amorphous hump with notable peaks, which corresponds to calcite (CaCO_3_: PDF #5-586), akermanite (Ca_2_MgSi_2_O_7_: PDF #35-592) and gehlenite (Ca_2_Al_2_SiO_7_: PDF #35-775).

As shown in Figure 6, CSH and CASH gels are detected in all of the AAS pastes. CSH gel is the main product of S5, which is consistent with the previous studies [13,40]. New characteristic peaks appear in S6, S7, and S8, when the activators of NaCl, CaCl_2_ and CaSO_4_ are added, respectively. When NaCl or CaCl_2_ is added, the characteristic peaks of Friedel’s salt (Fs salt, 3CaO⋅Al_2_O_3_⋅CaCl_2_⋅10H_2_O: PDF #35-105) appeared. Although the hydration products of S6 and S7 were similar, the compressive strength of the latter is higher because of the addition of Ca^2+^ ions from CaCl_2_ [20]. When CaSO_4_ is added, the characteristic peaks of ettringite (3CaO⋅Al_2_O_3_⋅3CaSO_4_⋅32H_2_O: PDF #41-1451) appear. When CaCO_3_ is added, no new characteristic peak generates. It should be noted that the diffraction peaks of S3 (SR- activated GGBS pastes) are similar to those of S7, and the main products of S3 are CSH gel, CASH gel, Fs salt, and calcite. As is discussed above, SR contains CaSO_4_, and ettringite is one of the products of S8 (Ca(OH)_2_ + CaSO_4_-activated GGBS pastes). However, no ettringite peak is found in S3 in Figure 6. It may be because ettringite is destabilized in the presence of Cl^−^ ions while Fs salt is formed [41,42,43].

### 3.4. SEM

**Figure 7:**
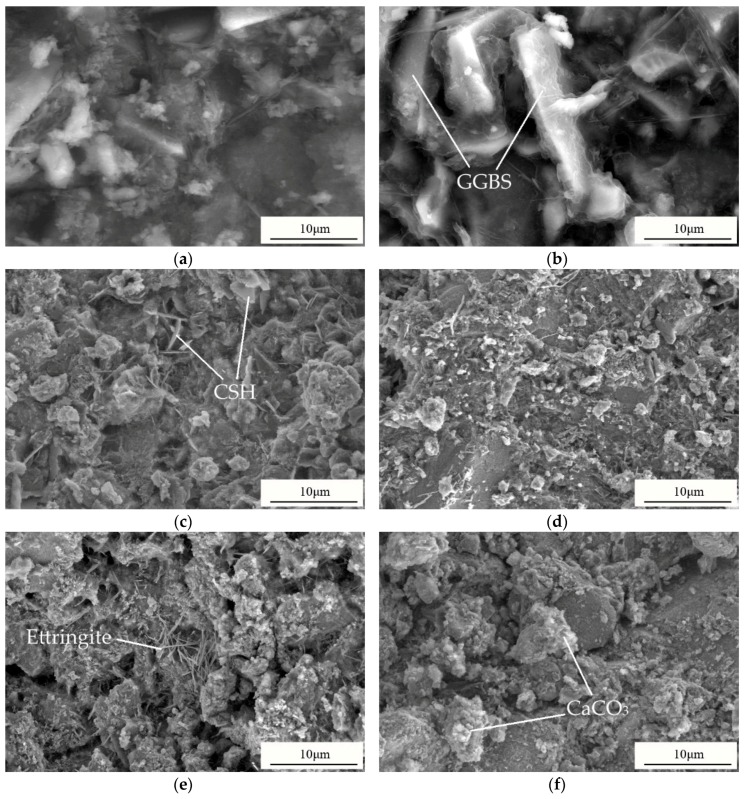
The SEM images of (**a**) S3; (**b**) S5; (**c**) S6; (**d**) S7; (**e**) S8; (**f**) S9.

SEM can detect most of the unhydrated phases, hydration products, and microstructures, which are the main factors influencing the strength. SEM images of AAS pastes (S3, S5, S6, S7, S8, S9) at 28 d are presented in Figure 7. It can be seen that the hydration products wrap the slag particles in all AAS pastes.

As shown in Figure 7, the GGBS particles in S5 can be seen clearly and are coated with CSH gel. Additionally, the microstructure of the pastes looks loose, and the hydration products are not enough to fill the pores among the particles, which results in low strength. In S6, flaky CSH gel can be found. The outline of GGBS particles can hardly be seen in S7, where the particles are surrounded by a large amount of gels, the bond between the particles is firm, and the structure is uniform and compact. Small-sized, needle-like ettringite appears in S8, which agrees with the XRD results. S9 exhibits denser microstructure, without any significant different products or amorphous phases, which is because CaCO_3_ fills the pores among the particles. It can be seen from Figure 7a that a large amount of products, amorphous phases, and CaCO_3_ fill in among the particles, making the structure of S3 more compact. No ettringite is found in S3, which is consistent with the XRD results. Therefore, S3, S5, S7, and S8 will be selected for FTIR analysis.

### 3.5. FTIR

**Figure 8:**
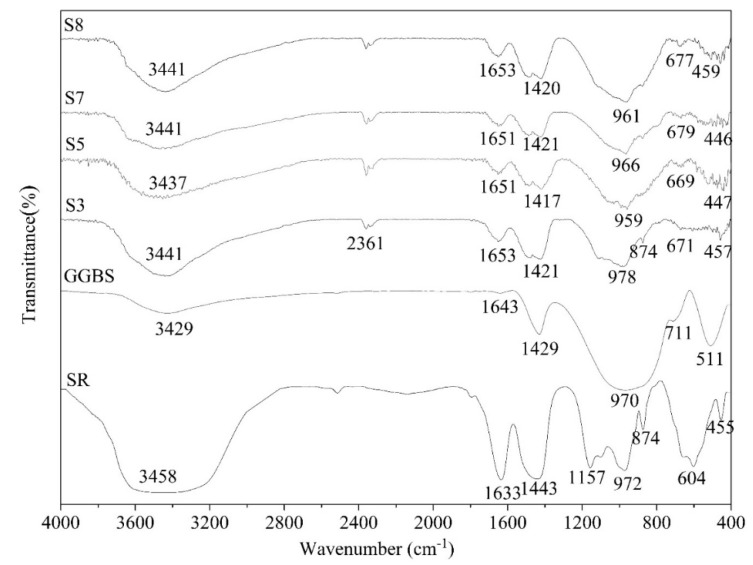
FTIR spectra of raw SR, raw GGBS, S3, S5, S7, and S8.

Fourier transform infrared spectroscopy (FTIR) is the key technology to further analyze the hydration products of AAS pastes. It can identify functional groups and compounds through the vibration of chemical bonds at the characteristic frequency. Figure 8 shows the FTIR spectra of the raw materials (SR, GGBS) and AAS pastes (S3, S5, S7, S8) at 28 d. All infrared band assignments given in this study are consistent with previous studies [14,20,44,45,46].

The vibrational bands among 3429 to 3458 cm^−1^, and at 2361 cm^−1^ are due to stretching vibrations of -OH and H-O-H bonds. In addition, the vibrational bands among 1633 to 1653 cm^−1^ correspond to the in-plane bending vibrations of H-O-H bonds. These absorption bands are due to crystalline H_2_O of the hydration products, and the free water molecules absorbed in the raw materials and AAS pastes, which agrees to previous studies [14,44]. The O-C-O stretching vibrational bands among 1417–1443 cm^−1^, and at 874 cm^−1^ correspond to the presence of CaCO_3_ phases [47,48], which indicates that carbonation is common in all AAS pastes. S3 shows absorption peak at 874 cm^−1^, due to the CaCO_3_ phases of SR. In SR, the O-S-O asymmetric stretching band at 1157 cm^−1^ can be clearly observed indicating that SR has CaSO_4_ phases, which is in agreement with the SEM and XRD results. The asymmetric stretching vibrational band at 970 cm^−1^ of raw GGBS assigned to Al (Si)-O-Si bonds shifts to lower frequencies (959, 966, and 961 cm^−1^) after alkaline activation, which indicates that the glassy structure of the GGBS dissolved under the alkaline conditions and Ca^2+^ ions participate in the condensation process to form CSH gel. The bands attributed to symmetric stretching vibration of Al-O-Si and out-of-plane bending vibration of Si-O-Si shift to lower frequencies from 711 cm^−1^ to 669–679 cm^−1^, and 511 cm^−1^ to 446–459 cm^−1^, respectively, indicating the formation of aluminum-silicate gels. With Ca(OH)_2_, CaCl_2_, and CaSO_4_ in SR, the Al (Si)-O-Si asymmetric stretching vibration band of S3 should shift to lower wavenumber such as S5, S7, and S8, but it shifts to higher wavenumber from 970 cm^−1^ to 978 cm^−1^. The most reasonable explanation is that the band at 978 cm^−1^ is an overlapping band of SO_4_^2−^ (1157 cm^−1^) and CSH gel (959–966 cm^−1^). As shown in Equation (2), Cl^−^ ions interact with monosulfate (3CaO⋅Al_2_O_3_⋅CaSO_4_⋅12H_2_O) to form Fs salt (3CaO⋅Al_2_O_3_⋅CaCl_2_⋅10H_2_O) [41,49]. This is the reason why no ettringite is generated in S3, which is consistent with XRD results.

(2) $$3\mathrm{CaO}\cdot {\mathrm{Al}}_{2}O_{3}\cdot {\mathrm{CaSO}}_{4}\cdot 12H_{2}O+2{\mathrm{Cl}}^{-}\to 3\mathrm{CaO}\cdot {\mathrm{Al}}_{2}O_{3}\cdot {\mathrm{CaCl}}_{2}\cdot 10H_{2}O+{\mathrm{SO}}_{4}^{2-}+2H_{2}O$$

## 4. Conclusions

This paper studies the behavior of SR-activated GGBS with different proportion of SR, and investigates the contribution of Ca(OH)_2_, NaCl, CaCl_2_, CaSO_4_, CaCO_3_ in SR to SR-activated GGBS pastes. The investigation is made using setting time and compressive strength testing, XRD, SEM, and FTIR techniques. Based on the obtained results, the following conclusions are made.

(1) The SR (24% addition)-activated GGBS pastes possess an acceptable setting time, high compressive strength (29.6 MPa) at 28 days, and the maximum utilization of solid waste SR. The results open the possibility to a massive use of SR in civil engineering.
(2) CaCl_2_ in SR plays a major role in causing short setting time, high compressive strength, more hydration products of SR-activated GGBS pastes. NaCl and CaSO_4_ in SR has a limited effect on setting time and compressive strength of SR-activated GGBS pastes. CaCO_3_ in SR is the main factor causing lower early compressive strength.
(3) The main hydration products of SR (24% addition)-activated GGBS pastes are CSH gel, CASH gel, and Fs salt, which ensures good mechanical properties of SR (24% addition)-activated GGBS pastes.

## Figures and Tables

**Table 1:** Chemical compositions of SR and GGBS used in this study (wt.%).

Components	CaCO_3_	CaCl_2_	Ca(OH)_2_	CaSO_4_	MgO	SiO_2_	NaCl	Al_2_O_3_	Others
**SR**	39.6	13.4	11.2	9.8	7	6.5	6	2	4.5
**Components**	CaO	SiO_2_	Al_2_O_3_	MgO	Fe_2_O_3_	TiO_2_	Na_2_O	K_2_O	Others
**GGBS**	41.4	28.1	14.8	9.5	1.4	1.1	0.6	0.6	2.5

**Table 2:** The calculation results and the mixing proportions of AAS cementitious materials (g).

Samples No.	GGBS	SR	Ca(OH)_2_	NaCl	CaCl_2_	CaSO_4_	CaCO_3_	H_2_O
S1	920.0	80.0	−	−	−	−	−	500.0
S2	840.0	160.0	−	−	−	−	−	500.0
S3	760.0	240.0	−	−	−	−	−	500.0
S4	680.0	320.0	−	−	−	−	−	500.0
S5	760.0	−	26.9	−	−	−	−	393.5
S6	760.0	−	26.9	14.4	−	−	−	400.7
S7	760.0	−	26.9	−	32.2	−	−	409.6
S8	760.0	−	26.9	−	−	23.5	−	405.2
S9	760.0	−	26.9	−	−	−	95.0	441.0

Note: All specimens were curing at room temperature in sealed plastic bags to avoid the loss of water.
